# Oral squamous cell carcinoma involving the maxillae: Factors affecting local recurrence and the value of salvage treatment for overall survival

**DOI:** 10.1002/hed.26108

**Published:** 2020-02-26

**Authors:** Fons J. B. Slieker, Remco de Bree, Ellen M. Van Cann

**Affiliations:** ^1^ Department of Head and Neck Surgical Oncology UMC Utrecht Cancer Center, University Medical Center Utrecht Utrecht The Netherlands

**Keywords:** 5‐year local control, malignancy, maxillary cancer, maxillectomy, salvage treatment, squamous cell carcinoma

## Abstract

**Objective:**

To determine factors associated with local recurrence (LR) of oral squamous cell carcinoma involving the maxillae (MSCC) and overall survival (OS) after salvage treatment.

**Subjects and Methods:**

Retrospective study of MSCC operated between 2000 and 2015. Kaplan‐Meier survival and Cox regression were used for analysis of MSCC‐associated clinical and histopathological factors.

**Results:**

Ninety‐five patients were included. LR occurred in 24% of patients. Vascular invasion significantly increased the risk of LR (hazard ratio 4.595, *P* = .003). Local salvage surgery, in the area of the original tumor, significantly prolonged OS, compared to palliative treatment (*P* = .001) and extensive salvage surgery (*P* = .013). Extensive salvage surgery, requiring resection of adjacent facial structures, did not prolong OS compared to palliative treatment (*P* = .186).

**Conclusions:**

MSCC with vascular invasion has higher risk of LR. Salvage surgery may prolong OS in small recurrences but might have dubious value for larger recurrences infiltrating adjacent facial structures.

## 1 INTRODUCTION

The preferred treatment for most malignant tumors involving the maxillae is surgery. The surgical approach and extent of the resection depend on the site and size of the tumor. Complete surgical removal of the tumor is critical as compromised margins impair the prognosis.^1^ Surgical removal of maxilla tumors can be technically challenging because certain areas are difficult to access, and the visibility may be poor. At the same time, vital structures near the tumor should be preserved. Local recurrence (LR) after maxillectomy is in part due to the relative inaccessibility of cranial and dorsal margins. Knowledge of other risk factors may help to early detect LR.

There is no consensus on the optimal salvage treatment strategy for recurrent tumors involving the maxillae. Salvage surgery is often the treatment of choice, but it is frequently at the cost of morbidity and quality of life.^2^ Insight into the overall survival rates of patients who have had salvage treatment of recurrent tumors involving the maxillae, might provide better information for physicians and patients, which may improve decision making.

The aim of this study is to identify factors associated with increased risk of LR after surgical treatment of oral squamous cell carcinoma involving the maxillae (MSCC) and to identify factors associated with decreased overall survival (OS) of salvage treatment of locally recurrent MSCC.

## 2 SUBJECTS AND METHODS

This study was granted an exemption from formal ethics review in writing by the “Institutional Review Board Utrecht,” because of its retrospective nature. Inclusion criteria were patients with MSCC, originating from the mucosa located on the alveolar process of the maxilla or the hard palate, operated between 2000 and 2015. Patients with second primary MSCC or sinonasal tumors were excluded.

### 2.1 Data collection

The following data were collected from medical records: date of birth, sex, alcohol and tobacco use, tumor location, tumor histology, type of surgery, operation date, pathological tumor stage, resection margins, spider growth pattern, nerve invasion, vascular invasion, bone invasion, LR, date of LR diagnosis, location of LR, (extent of) salvage treatment, palliative treatment, and date of death.

### 2.2 Preoperative screening

Preoperative screening consisted of physical examination, orthopantomogram, MRI‐scan and/or CT‐scan, chest X‐ray, and ultrasound of the neck with fine‐needle aspiration cytology on indication. The seventh edition of the T/N/M classification was used for staging.^3^ All patients were discussed in a weekly multidisciplinary team meeting and treated according to the national guidelines (https://richtlijnendatabase.nl/richtlijn/hoofd-halstumoren/hoofdhalstumoren_-_korte_beschrijving.html).

### 2.3 Surgery

Surgery was performed within 4 weeks from presentation in the outpatient department. Surgery included local excision, partial maxillectomy, hemimaxillectomy, or (sub)total maxillectomy. The surgical defects were managed with secondary wound healing, local flaps, free flaps, or obturator prostheses.

### 2.4 Primary treatment of the neck

Patients with clinically positive lymph nodes were treated as a rule by neck dissection. A few patients received primary radiotherapy of the neck instead of neck dissection for patient‐specific reasons.

### 2.5 Histology

The resection specimens were histologically examined. Data items included in the histopathology report of the resection specimen were histological cell type, tumor size, infiltration depth, resection margins (<1 mm was considered positive^4^), spider growth pattern, nerve invasion, vascular invasion, and bone invasion. Data items included in the histopathology report of the neck dissection specimen were number, size and site of metastatic lymph‐nodes, and presence of extracapsular spread.

### 2.6 Adjuvant treatment for high risk factors

Positive surgical margins were managed by re‐excision if possible, preferably when the temporary obturator prostheses was adjusted after 2‐3 weeks; if re‐excision was not possible then postoperative radiotherapy was applied. Postoperative radiotherapy was also applied for extracapsular spread. Since 2005, chemotherapy was added to radiotherapy in patients <70 years with positive surgical margins and/or extracapsular spread without contraindications for chemotherapy.

### 2.7 Adjuvant treatment for intermediate risk factors

Postoperative radiotherapy was applied when three or more intermediate risk factors were present for recurrence, that is, close resection margins, nerve invasion, pT3/T4 tumors, and/or multiple positive lymph nodes. Postoperative radiotherapy was started within 6 weeks of surgery.

### 2.8 Follow‐up

Follow‐up appointments were scheduled every 2 months in the first postoperative year, every 3 months in the second year, every 4 months in the third year, every 6 months in the fourth year and fifth year. Patients free of disease after 5 years were discharged from follow‐up.

### 2.9 Salvage treatment

Patients presenting with LR were considered for salvage surgery. Salvage surgery was classified as local salvage surgery when confined to the area of the original tumor. Salvage surgery was classified as extensive salvage surgery when requiring resection of adjacent facial structures (eg, zygomatic resection, enucleation). Palliative treatment with (chemo)radiotherapy was offered for irresectable LR or when the patient declined surgery.

### 2.10 Definitions

The 5‐year local control rate was defined as the proportion of patients without LR in the area of the original primary tumor within 5 years after surgery.

### 2.11 Analysis

The location of LR was listed to identify areas at risk for LR.

Kaplan‐Meier survival analysis^5^ was used to calculate the 5‐year local control rate of MSCC. The log rank test (*α* = .05) was conducted to analyze differences between groups.

Cox regression analysis was conducted to calculate whether clinical or histopathological factors were associated with the likelihood of 5‐year LR.

Kaplan‐Meier and Cox regression analyses were also used to analyze factors affecting OS after salvage treatment of locally recurrent tumors.

The following results of the regression analyses were listed: *P* value, hazard ratios (HRs), and 95% confidence intervals (CIs). Independent variables were considered statistically significant when *P* < .05. Missing data were handled by pairwise deletion.

Analysis was aided by the Statistical Package for the Social Sciences (version 25.0 for Windows, SPSS Inc., Chicago, IL) and guided by Laerd statistics.^6^

## 3 RESULTS

Between 2000 and 2015, 128 consecutive patients had been operated for malignant tumors of the maxilla. Of these 128 patients, 95 had MSCC tumors and were included. The patient characteristics are listed in Table 1.

**Table 1:** Pertinent clinical and histopathological data

Patient characteristics	Total (n = 95)
*Sex*	
Male	41
Female	54
*Median age in years (lowest to highest)*	
Male	69 (46‐93)
Female	71 (43‐96)
*Tumor location*	
Alveolar process	74
Hard palate	21
*cT‐stage*	
cT1‐2	45
cT3‐4	50
*Treatment*	
Surgery	57
Surgery + (chemo)radiotherapy	38
*pT‐stage*	
pT1‐2	44
pT3‐4	51
*Surgical margins*	
Clear (≥1 mm)	56
Positive (<1 mm)	39
*Bone invasion*	
Absent	34
Present	61
*Spider growth pattern*	
Absent	61
Present	34
*Nerve invasion*	
Absent	79
Present	16
*Vascular invasion*	
Absent	87
Present	8
*5‐y LR*	
Sisease free	72
Locally recurrent disease	23

Abbreviation: LR, local recurrence.

**Figure 1:**
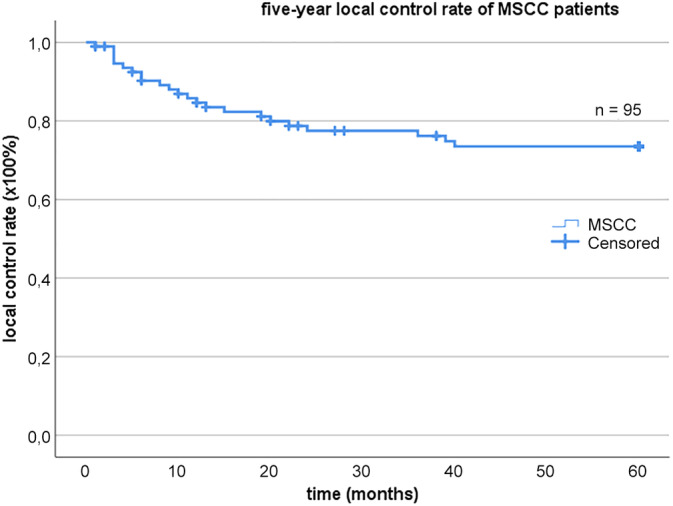
Kaplan‐Meier curve with five‐year local control rate of MSCC patients. MSCC, cell carcinoma involving the maxillae [Color figure can be viewed at wileyonlinelibrary.com]

In total, 23 out of 95 (24%) patients developed LR. The mean time of diagnosis of LR was 12 months (range 1‐40 months) after primary treatment. At the 5‐year endpoint, the local control rate of the MSCC group was 76% (Figure 1 for the Kaplan‐Meier survival analysis).

### 3.1 Factors associated with 5‐year LR of MSCC

**Figure 2:**
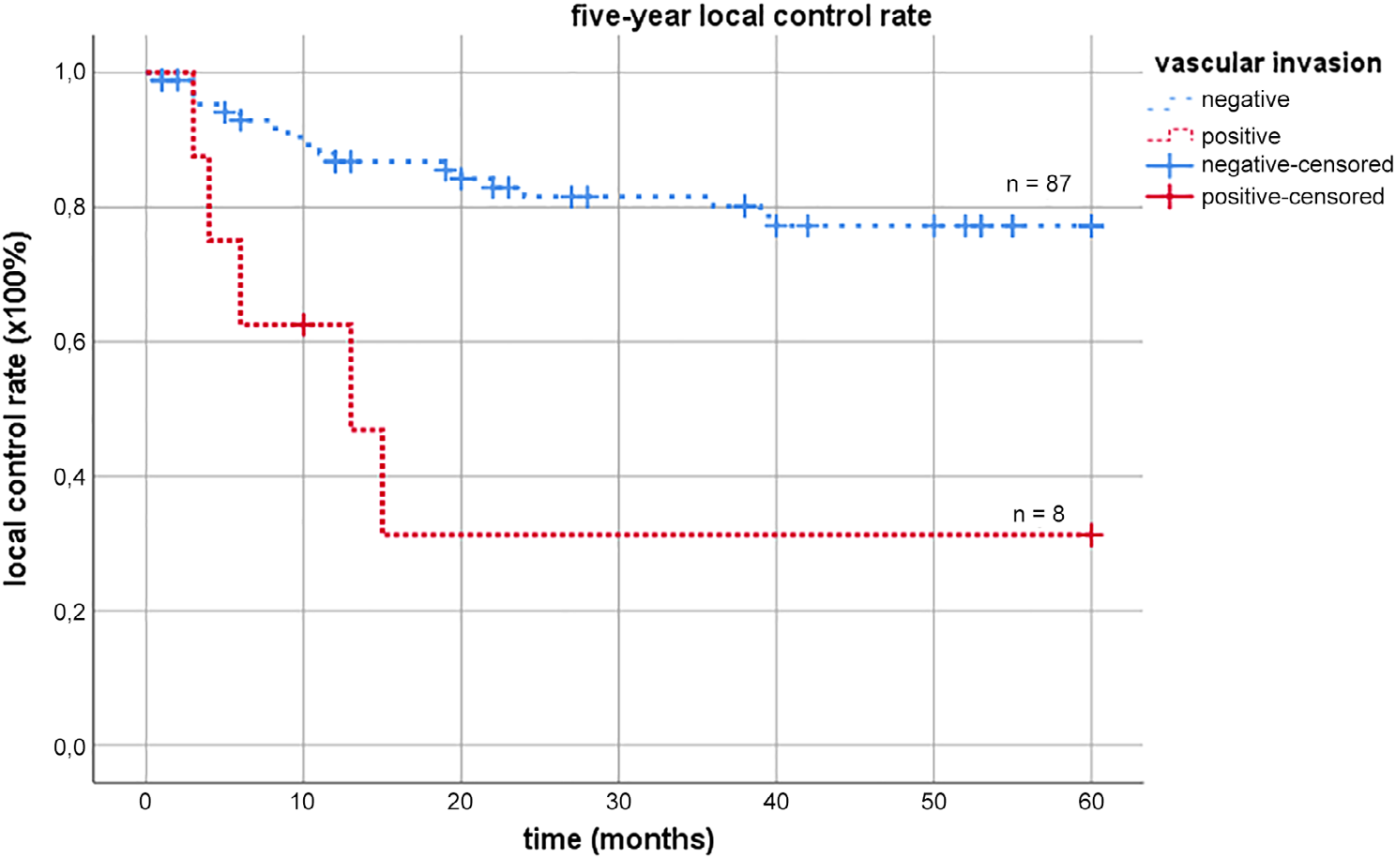
Kaplan‐Meier curve with 5‐year local control rate of MSCC with vascular invasion vs no vascular invasion. MSCC, cell carcinoma involving the maxillae [Color figure can be viewed at wileyonlinelibrary.com]

Cox regression analyses showed that vascular invasion (HR 4.595, 95% CI [1.683‐12.543], *P* = .003) was significantly associated with the likelihood of LR within 5 years after surgery (Table 2). In this cohort, six out of eight patients with vascular invasion were diagnosed with LR within 15 months after surgery (Figure 2).

**Table 2:** Univariate cox regression analyses of factors potentially associated with 5‐year LR of MSCC

Univariate Cox proportional hazard	*P*	Hazard ratio	Odds ratio (95% CI)	
Sex	.399	.691	.293	1.630
Age	.248	1.021	.986	1.057
Tumor location	.548	.718	.244	2.113
cT‐stage (T3‐4 vs T1‐2)	.815	.907	.400	2.057
Treatment of primary tumor	.394	.680	.279	1.654
pT‐stage (T3‐4 vs T1‐2)	.289	1.574	.680	3.643
Surgical margins (positive vs clear)	.414	1.412	.617	3.230
Bone invasion	.069	2.511	.930	6.780
Spider growth pattern	.872	1.073	.454	2.536
Nerve invasion	.599	1.336	.454	3.937
Vascular invasion	**.003**	4.595	1.683	12.543

Abbreviations: CI, confidence interval; LR, local recurrence; MSCC, cell carcinoma involving the maxillae.

Bold values are statistically significant.

### 3.2 Location of LR

In 17 out of 23 cases, LR emerged at the dorsal margin, either dorsocranial or dorsocaudal (Table 3). LR at dorsocranial margins extended into the maxillary sinus, nasal cavity, orbital complex, sphenoid bone, ethmoid bone, pterygoid process, and/or intracranially (carotid groove, meninges, and subarachnoid space of frontal lobe). LR at the dorsocaudal margin extended into the soft palate, hypopharynx, retropharyngeal and parapharyngeal space or encased the internal carotid artery. In 4 out of 23 cases, LR was located at the lateral margin, involving the buccal mucosa. In 2 out of 23 cases, LR was located superficially at the mucosal surface of the resected primary tumor.

**Table 3:** Local recurrences: time to LR (months), site, compromised margin, type of salvage treatment, and survival time after salvage treatment

Patient	Time to LR (mo)	LR location	Compromised margins	Treatment of LR	Survival after salvage (mo)
1	*24*	Left maxillary sinus, pterygopalatine fossa, buccal mucosa, orbit and ear	Dorsocranial	Palliative treatment	180
2	6	Orbit	Dorsocranial	Palliative treatment	4
3	8	Left maxillary sinus, infratemporal fossa, orbit and anterior subcutis	Dorsocranial	Palliative treatment	5
4	1	Right nasal cavity	Dorsocranial	Palliative treatment	1
5	3	Cavernous sinus, orbit, infra temporal fossa, sphenoid sinus, and temporal lobe	Dorsocranial	Palliative treatment	5
6	3	Right zygomatic bone, orbit, palate, and parapharyngeal space	Dorsocranial and dorsocaudal	Right enucleation and partial zygomatic resection	7
7	10	Left maxillary sinus, orbit, zygomatic bone, concha inferior, and soft palate	Dorsocranial and Dorsocaudal	Right hemimaxillectomy, enucleation, and zygomatic resection	11
8	4	Left buccal mucosa	Lateral	Palliative treatment	2
9	6	Right maxillary sinus, buccal mucosa, orbital surface, and concha media	Dorsocranial and lateral	Enucleation, buccal resection, and hemirhinectomy	5
10	13	Upper left incisor, alveolar process, and buccal mucosa	Lateral	Partial maxillectomy	16
11	15	Left retropharyngeal space, total encasement of left internal carotid artery	Dorsocaudal	Palliative treatment	3
12	3	Right soft palate	Dorsocaudal	Palliative treatment	2
13	5	Right maxillary sinus, nasal floor, and retropharyngeal space	Dorsocranial and caudal	Palliative treatment	2
14	11	Retropharyngeal space, masticator space, carotid groove	Dorsocaudal	Palliative treatment	1
15	20	Right buccal mucosa	Lateral	Local resection	Alive after 36 mo
16	22	Soft palate	Dorsocaudal	Local resection	11
17	19	Right buccal mucosa	Lateral	Local resection	Alive after 13 mo
18	12	Dorsal maxillary bone invasion, buccal mucosa, mandible	Dorsocaudal	Palliative treatment	6
19	3	Left eustachian tube	Dorsocranial	Local resection	Alive after 193 mo
20	9	Medial hard palate	Local	Local resection	Alive after 30 mo
21	36	Right hard palate	Local	Local resection	13
22	40	Right hard palate and maxillary tuberosity with invasion of maxillary sinus	Dorsocranial	Partial maxillectomy	8
23	3	Right maxillary sinus	Dorsocranial	Palliative treatment	10

Abbreviation: LR, local recurrence.

### 3.3 Overall survival after salvage treatment of recurrent MSCC

Cox regression analyses demonstrated that the type of salvage treatment was significantly associated with the likelihood of OS after salvage treatment (*P* = .009) (Table 4). The presence of bone invasion (*P* = .056) and LR localization (*P* = .083) approached a statistically significant association with the likelihood of OS. Previous treatment of the primary tumor, time interval to LR, surgical margins after salvage surgery, spider growth pattern, vascular invasion, and perineural invasion of the recurrent tumor were not associated with the likelihood of OS after salvage treatment (all *P* ≥ .348).

**Table 4:** Univariate Cox regression analyses of factors potentially associated with OS after salvage treatment of locally recurrent MSCC

Univariate Cox proportional hazard	*P*	Hazard ratio	Odds ratio (95% CI)	
Treatment of primary tumor	.327	1.613	.620	4.194
Time interval to LR (<6 mo vs ≥6 mo)	.348	1.556	.618	3.919
LR localization	.083	‐	‐	‐
Salvage treatment type	**.009**	‐	‐	‐
Surgical margins after salvage (positive vs clear)	.799	1.238	.238	6.430
Bone invasion	.056	10.634	.940	120.341
Spider growth pattern	.689	1.362	.299	6.198
Nerve invasion	.531	2.018	.225	18.114
Vascular invasion	.636	1.670	.200	13.934

Abbreviations: CI, confidence interval; LR, local recurrence; MSCC, cell carcinoma involving the maxillae; OS, overall survival. The bold *P*‐value statistically significant

In this study, the salvage treatment types were classified as palliative treatment, local salvage surgery, and extensive salvage surgery.

Six out of 23 LR cases underwent local salvage surgery, 5 out of 23 LR cases underwent extensive salvage surgery with resection of adjacent structures (orbit, ethmoid, zygoma, the other half of maxilla, or external nose) and 12 out of 23 LR cases received palliative treatment (Table 3).

**Figure 3:**
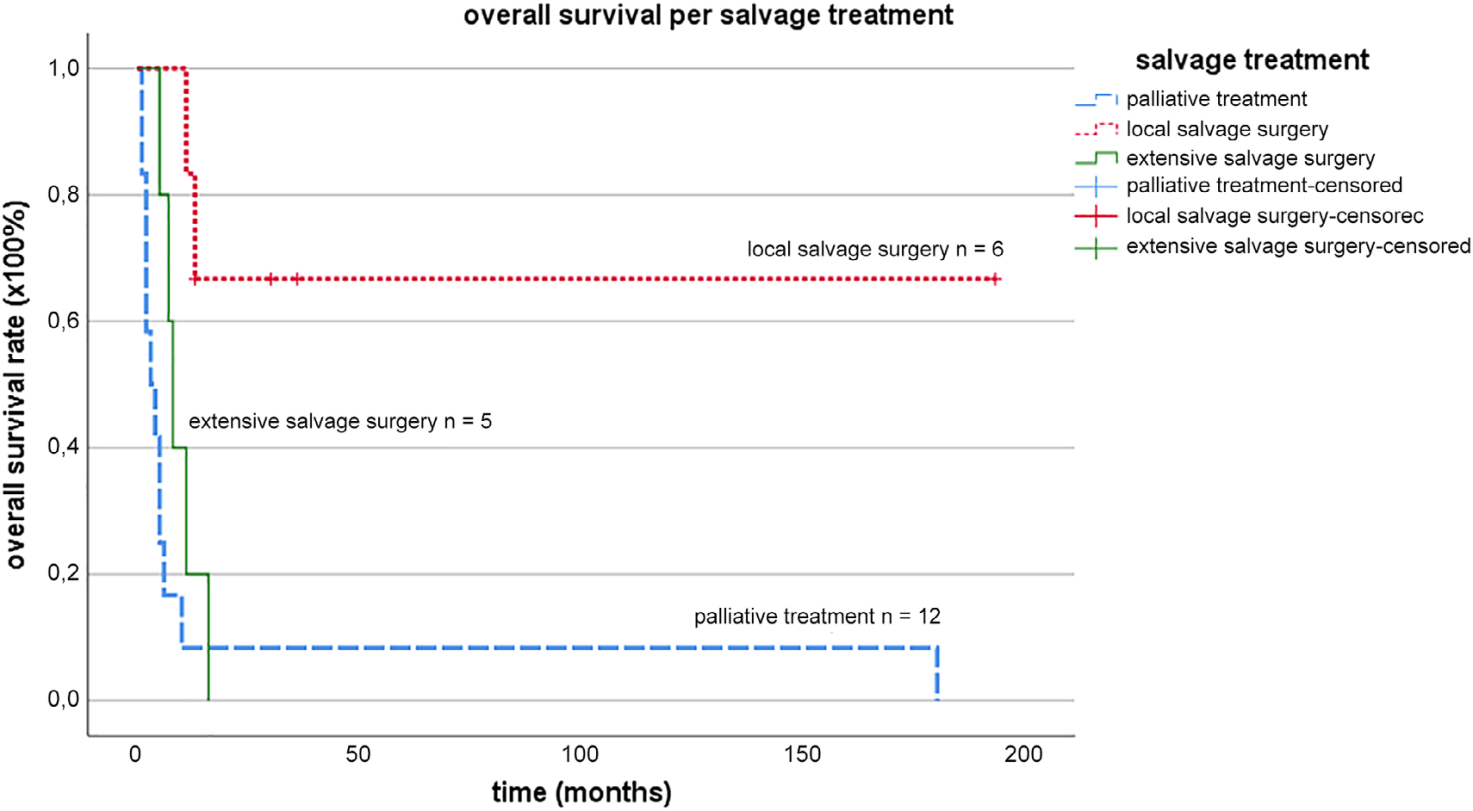
Kaplan‐Meier curves with overall survival rates after salvage treatment of recurrent MSCC. MSCC, cell carcinoma involving the maxillae [Color figure can be viewed at wileyonlinelibrary.com]

The Kaplan‐Meier survival analysis of these three salvage treatment groups is displayed in Figure 3. From the extensive salvage surgery group, 5 out of 5 patients (100%) died and from the palliative treatment group, 12 out of 12 patients (100%) died. Patients who received palliative treatment had a median survival time of 3.0 months (95% CI [0‐6.4]), which was not significantly different (*χ* ^2^ = 1.753, *P* = .186) from the median survival time of patients who had extensive salvage surgery: 8.0 months (95% CI [5.8‐10.1).

Four out of 6 patients (80%) from the local salvage surgery group were still alive at the time of this study. One deceased patient who had had local salvage surgery after 10 months, and the other died after 13 months. OS after local salvage surgery was significantly longer than OS after palliative treatment (*χ* ^2^ = 10.270, *P* = .001) and longer than OS after extensive salvage surgery (*χ* ^2^ = 6.174, *P* = .013).

## 4 DISCUSSION

Vascular invasion was significantly associated with an increased likelihood of LR, even though there were only eight patients with vascular invasion in this cohort. In the literature, LR has been associated with positive surgical margins, T3‐4 stage, dorsocranial tumor extension, and nerve invasion, but to our knowledge not with vascular invasion.

LR occurred most frequently at the dorsal margins (cranial/caudal). A possible explanation for the occurrence of LR at the dorsal margins is the difficulty to achieve tumor free resection margins at these distant locations.^7^ Another explanation for the occurrence of LR in the posterior region is that occult metastases may develop in the upper jugular nodes and/or lateral retropharyngeal nodes. These nodes are not routinely removed during the primary surgical treatment when they seem uninvolved during the preoperative screening, but they may develop occult metastasis.^8^, ^9^ To reduce the risk of recurrent disease developing from these nodes, Tiwari et al^8^ and Yanamoto et al^10^ recommend en‐bloc maxillectomy and internal dissection of the masticator space through a transmandibular approach.

### 4.1 Treatment of LR

To the best of our knowledge, this is the first study to analyze factors potentially associated with the likelihood of OS after salvage treatment. The type of salvage treatment was significantly associated with the likelihood of OS.

OS after local resection of recurrent tumors was longer than OS after palliative treatment or OS after extensive salvage surgery. Extensive salvage surgery had no survival advantage over palliative treatment. Our results suggest that extensive salvage surgery should be considered with caution, as its value in terms of OS may be dubious. It should be considered that these extensive procedures may disturb the appearance and function while quality of life is particularly important in the final period of life.

### 4.2 Limitations

A limitation of this study was its retrospective study design. Risk of information bias is possible, because data was collected from medical records which were recorded by several physicians in a period of 18 years.

Furthermore, the seventh edition of the T/N/M classification had to be used, because data on tumor infiltration depth was not retrievable for older cases, which made reclassification according to the eighth edition of T/N/M classification unsuitable. Future studies about the effects of infiltration depth and T/N/M classification differences of MSCC are therefore of interest.

## 5 CONCLUSIONS

LR occurred in 24% of patients. Patients with MSCC and vascular invasion are at risk for LR. Salvage surgery prolongs OS in case of small recurrences but might have dubious value regarding OS for larger recurrences infiltrating adjacent facial structures.

## CONFLICT OF INTEREST

The authors declare no potential conflict of interest.
